# Supplementary material for: Exchange pathways of plastoquinone and plastoquinol in the photosystem II complex
Source: Nat Commun. 2017 May 10;8:15214. doi: 10.1038/ncomms15214 (PMC5436218; doi:10.1038/ncomms15214)
Supplement: Supplementary Information — Supplementary Figures, Supplementary Tables, Supplementary Note, Supplementary Methods and Supplementary References [file ncomms15214-s1.pdf]

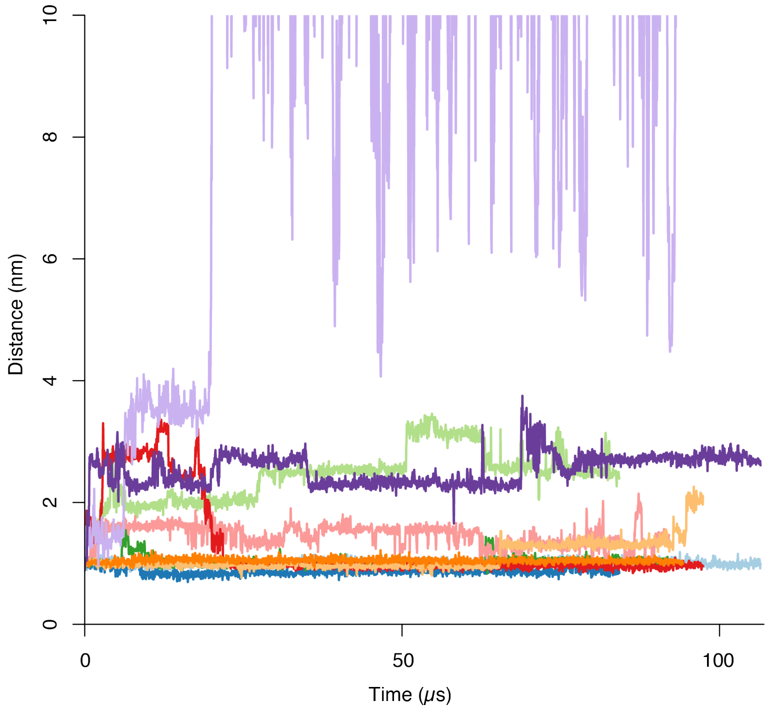

Supplementary Figure 1: Unbinding traces of PLQols out of the Q_B site. Distance between the centre of mass of the PLQol headgroup and the non-heme iron is shown over time. Each color represents one PLQol molecule. Out of the ten molecules (5 simulations, 2 per monomer), five PLQol molecules leave the binding pocket. One of the PLQols (pink trace) ends up in the thylakoid membrane, the others remain trapped in the PLQ exchange cavity or stuck to the side of the protein.

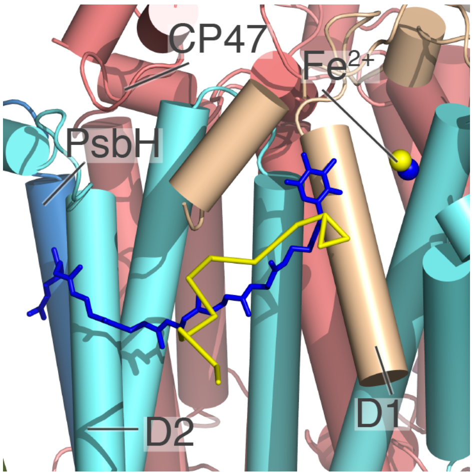

Supplementary Figure 2: Binding pose of PLQ at the Q_B binding site. Positions of Q_B in the crystal structure (blue) and the best binding PLQ in the adaptive CG MD simulation (yellow). The position of the non-heme iron in the respective structures is also indicated.

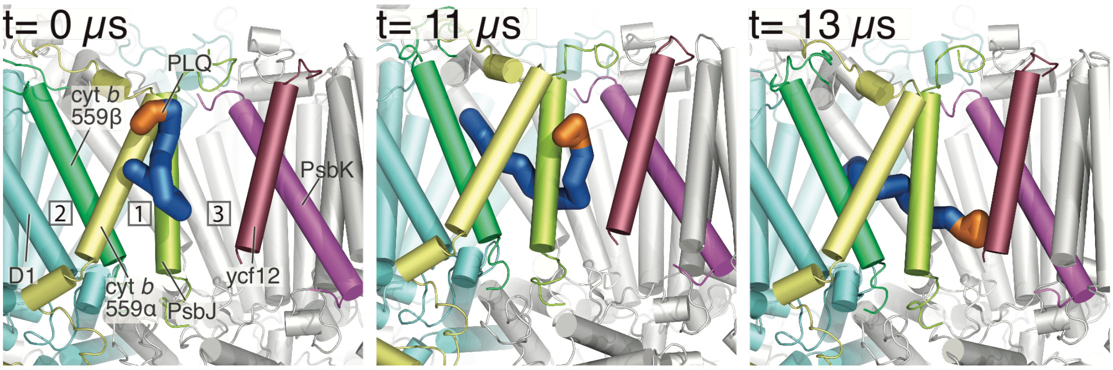

Supplementary Figure 3: PLQ can enter tail first. Snapshot showing the tail first entry of a particular PLQ through channel III. Reorientation occurs later inside the exchange cavity. PLQ is colored blue, with its headgroup in orange for clarity. Helices are colored as in Figure 1b.

Supplementary Table 1: Residues[a] lining the channels and the cavity.

Channel	Wall 1	Wall 2
I	PsbJ-TRP11 – PsbJ-LEU36	Cyt b 559 α -SER16 – Cyt b 559 α -TRP35 and Cyt b 559 β -TYR13 – Cyt b 559 β -PHE32
II	Cyt b 559 α -SER16 – Cyt b 559 α -TRP35 and Cyt b 559 β -TYR13 – Cyt b 559 β -PHE32	D2-PHE27 – D2-THR53
III	Ycf12-VAL18 – Ycf12-PHE37	PsbJ-TRP11 – PsbJ-LEU36
Cavity center residues		
D1-SER268 – D1-ALA294		

[a]Of some residues not all beads were included: D1-SER268 only SC1, D2-PHE27 only SC2 and SC3, Cyt b 559 α -TRP35 only the backbone bead (BB), Cyt b 559 β -TYR13 only beads SC1, SC2 and SC3, PsbJ-TRP11 only the SC2, SC3 and SC4 bead, PsbJ-LEU36 only the BB and ycf12-PHE37 only the BB.

Supplementary Table 2: Number of PLQs and PLQols diffusing in and out of the PLQ exchange cavity through the three channels[a].

	Channel											
	Left monomer						Right monomer					
	I		II		III		I		II		III	
Simulation	IN	OUT	IN	OUT	IN	OUT	IN	OUT	IN	OUT	IN	OUT
I	**1**+1	**0**+1	**0**+0	**0**+1	**1**+2	**0**+2	**1**+0	**0**+0	**0**+1	**0**+0	**1**+2	**1**+2
II	**1**+0	**0**+0	**0**+3	**0**+1	**1**+3	**1**+3	**1**+1	**0**+1	**0**+0	**0**+0	**1**+3	**1**+3
III	**2**+8	**3**+8	**0**+1	**0**+1	**1**+4	**0**+4	**2**+2	**1**+2	**0**+1	**0**+1	**0**+0	**0**+0
IV	**0**+0	**0**+0	**0**+2	**0**+2	**2**+0	**2**+0	**1**+2	**0**+2	**0**+1	**0**+0	**0**+2	**0**+2
V	**0**+0	**0**+0	**1**+1	**1**+1	**1**+0	**0**+0	**1**+0	**0**+0	**0**+0	**0**+0	**0**+0	**1**+0
sum	**4**+9	**3**+9	**1**+7	**1**+6	**6**+9	**3**+9	**6**+5	**1**+5	**0**+3	**0**+1	**2**+7	**3**+7

channel	I (IN)	I (OUT)	II (IN)	II (OUT)	III (IN)	(III OUT)
sum over dimer	**10**+14	**4**+14	**1**+10	**1**+7	**8**+16	**6**+16

[a]In **bold** are the cofactors that completely diffuse through a channel, while the numbers after the '+' represent the number of cofactors that still remain somehow attached or inside the PLQ channel and do not fully enter or leave the PLQ exchange cavity. The numbers are split out over the five independent simulations, but sums are given as well. Aggregated simulation time is about 0.5ms.

Supplementary Table 3: Comparison of the flux[a] of PLQol through different channels.

	Channel I	Channel II	Channel III
**Flux in**	10 ± 2	0 ± 0	2 ± 1
**Flux out**	6 ± 2	0 ± 0	2 ± 1

[a]The average flux of PLQols [# molecules/ms/monomer], diffusing in and out of the two PLQ exchange cavities through the three channels, is shown. Standard errors are given based on twelve independent measurements (six simulations, two monomers). More details are shown in Supplementary Table 4.

Supplementary Table 4: Number of PLQols diffusing in and out of the PLQ exchange cavity through the three channels in the simulations with PLQol added[a].

	Channel											
	Left monomer						Right monomer					
	I		II		III		I		II		III	
Simulation	IN	OUT	IN	OUT	IN	OUT	IN	OUT	IN	OUT	IN	OUT
I	0+0	0+0	0+2	0+0	0+0	0+0	**1**+2	**1**+2	0+0	0+0	**1**+1	0+1
II	**2**+0	**1**+0	0+1	0+0	0+0	**1**+0	0+2	0+2	0+0	0+0	0+0	0+0
III	0+2	0+2	0+0	0+0	0+0	0+0	0+0	0+0	0+0	0+0	0+0	0+0
IV	0+0	0+0	0+1	0+1	0+0	0+0	0+0	0+0	0+0	0+0	0+0	0+0
V	0+0	0+0	0+0	0+0	0+1	0+1	0+0	0+0	0+0	0+0	0+0	0+0
VI	**1**+0	**1**+0	0+0	0+0	0+0	0+0	**1**+1	0+1	0+0	0+0	0+0	0+0
sum	**3**+2	**2**+2	0+4	0+1	0+1	**1**+1	**2**+5	**1**+5	0+0	0+0	**1**+1	0+1

channel	I (IN)	I (OUT)	II (IN)	II (OUT)	III (IN)	(III OUT)
sum over dimer	**5**+7	**3**+7	0+4	0+1	**1**+2	**1**+2

[a]In **bold** are the cofactors that completely diffuse through a channel, while the numbers after the '+' represent the number of cofactors that still remain somehow attached or inside the channel and do not fully enter or leave the PLQ exchange cavity. The numbers are split out over the six independent simulations, but sums are given as well. Aggregated simulation time is about 0.25ms.

Supplementary Note 1: Limitations of our approach

Molecular dynamics are a powerful tool to probe time and length scales that are experimentally difficult to access. They are however still based on models and are therefore prone to inaccuracies and biased by underlying assumptions. One of the basic assumptions underlying the use of a CG model is that some of the atomistic degrees of freedom are irrelevant in light of the questions being addressed, and can be pre-averaged out. Specific limitations pertaining to the CG Martini model that was used in our study are discussed in [1]. Of importance here are four issues in particular: (i) simplification of the aqueous phase and electrostatics, (ii) the use of an elastic network to stabilize protein conformations, (iii) modelling of ring structures, and (iv) interpretation of the time scale.

Concerning (i), the standard Martini water model as used in this study is a Lennard-Jones fluid with implicit electrostatic screening. We showed previously that, despite the simplistic description of the aqueous surrounding, our PSII complex is stable on time scale of our simulations, behaving qualitatively similar to what is observed in (shorter) atomistic studies - no artefacts due to the simplification of the solvent have been noticed [2]. The behavior of PLQ/PLQol is mainly governed by hydrophobic interactions, which are captured well at the Martini level. In a previous study we showed that the orientation and conformations of PLQ and PLQol in a bilayer closely mimics that of all-atom models [3]. More generally, the Martini model has proven to be capable of revealing specific protein-lipid interactions in many cases [4].

Concerning (ii), coarse-graining implies that the directionality of hydrogen bonds is lost. Secondary structure elements in the Martini model therefore require an auxiliary elastic network to remain close to their native fold. As long as one is not interested in folding and unfolding events, this assumption is fine. It is important to note that tertiary or quarternary structural rearrangements (e.g., the opening and closing of the PLQ exchange channels) can be adequately captured with the Martini model; many examples exist of protein-protein interactions that have been either validated by all-atom models or experimentally [1].

Concerning (iii), ring-like moieties, such as the PLQ head group, pose a challenge due to their anisotropic nature. Although the ring-like structure can be captured in the Martini model, the effective thickness of the rings is somewhat larger than in atomistic models. This could explain the kinetic trapping of PLQ and PLQol in channel II, the narrowest of the three channels.

Concerning (iv), smoothening of the underlying energy landscape as a result of using effective interactions results in reduced friction and hence increase in dynamics of the system. Although this is advantageous with respect to sampling efficiency, it implies that the interpretation of the time scale has to be done with care. Typically, speed-up factors of between 2 and 10 have been reported for the Martini model, depending, however, on system details in a non-obvious way. The time scales reported in the main paper should therefore be considered as order of magnitude estimates only.

Supplementary Methods

Definition of PLQol binding

For PLQol, leaving the Q_B binding site is defined as the moment the distance between the PLQol headgroup and the non-heme iron is larger than 1.5 nm. The distance between the PLQol headgroups and the non-heme iron were calculated using the Gromacs tool g_dist. For the PLQol headgroup the centre of mass of the three headgroup beads was used.

Visualization of diffusion pathways

PLQ diffusion pathways were visualized by calculating the time-average density of the cofactor throughout the simulation box. For this purpose all five simulations with PLQ in the thylakoid membrane were concatenated and fitted on the protein backbone using the Gromacs tool trjconv. The calculation of the density was done using the Volmap tool in VMD with the resolution set to 0.2 nm after fitting the simulation on the protein backbone (BB) bead [5]. The threshold for showing occupancies was set to give a clear, qualitative view of the channels, while still being able to distinguish from the bulk. The PLQ densities reveal clear pathways in the simulation box with a threshold value of 8% (implying that at least in 8% of the frames a PLQ bead is present, which corresponds to roughly 2.4 times the occupancy of PLQ in the bulk).

Calculation of fluxes

In order to count the flux of PLQs and PLQols through the three channels, first the residues lining the cavity were determined. For each channel we defined two ‘walls’ that line the channel, one on each side, see Supplementary Table 1. The diffusion of PLQ in or out of a channel can easily take several microseconds and due to its elongated nature it is possible for a PLQ to be present in two channels concurrently. Therefore the diffusion of PLQs was tracked on the level of the cofactor as a whole, as well as for each individual cofactor bead. For each frame in the simulation, we determined for each individual PLQ/PLOol bead if it resided within 0.6 nm of one of the walls. If a cofactor bead was in contact with two walls that form together a channel, the bead was assigned to that channel. In case it was in contact with three or more walls, it was assigned to the two walls with which it had the most contacts and provided that these two walls line the same channel, the bead was assigned to this channel. Once a bead had been assigned to a channel and in a subsequent frame loses contact with one or both of the walls, it was checked whether the bead resides inside or outside the PLQ exchange cavity. For this purpose a group of residues in the back of the PLQ exchange cavity was defined (cavity residues), see Supplementary Table 1. The PLQ bead was considered to be in the cavity when the distance between the center of geometry of the cavity residues and the bead was smaller than the distance between the center of geometry of the cavity residues and the center of geometry of the two channel walls. If the distance between the PLQ bead and the cavity residues was bigger than the distance between the cavity residues and the channel center of geometry, the bead was considered to be outside the protein. Once it had been determined for all the beads of a cofactor if they reside in or out of the PLQ exchange cavity, it was determined if the cofactor as a whole resides in or outside the PLQ cavity. In order to only count an entry or exit when the cofactors beads diffused

through the same channel, the status of a cofactor, as being in or outside the PLQ cavity, was updated only when more than 70% of the PLQ beads had diffused in or out of the PLQ cavity through the same channel. Each time a PLQ entered or left the cavity this was recorded, as well as which channel it used. This method, however, is not foolproof due to the movement of the channel walls. Therefore, the simulations were visually inspected to verify each crossing and to check for missed crossings. Flux analysis was implemented in the Python language, using the MDAnalysis package for handling of MD data [6,7]. Pictures were rendered in VMD and in Pymol [5,8], movies were rendered in VMD [5].

Determining channel sizes

Channel sizes were estimated by measuring the distance between the residues lining the channels in VMD [5]. The values presented in the main manuscript are averages, as the width of the channels varies across the membrane.

Simulation setup with excess PLQol

To study the exchange behaviour of PLQol in more detail, six replicate simulations were performed, in which not PLQ, but PLQol was added to the bilayer at 5 mol%. These had a length of 44.8 μs, 52.8 μs, 40.8 μs, 43.3 μs, 38.8 μs and 38.6 μs, totalling to more than 0.25 ms. Simulation conditions were otherwise identical to those described in the main paper.

Supplementary references

1. Marrink, S. J. & Tieleman, D. P. Perspective on the Martini model. *Chem. Soc. Rev.* **42,** 6801–6822 (2013).
2. van Eerden, F. J. *et al.* Molecular Dynamics of Photosystem II Embedded in the Thylakoid Membrane. *J. Phys. Chem. B* acs.jpcb.6b06865 (2016). doi:10.1021/acs.jpcb.6b06865
3. de Jong, D. H. *et al.* Atomistic and Coarse Grain Topologies for the Cofactors Associated with the Photosystem II Core Complex. *J. Phys. Chem. B* **119,** 7791–7803 (2015).
4. Hedger, G. & Sansom, M. S. P. Lipid interaction sites on channels, transporters and receptors: Recent insights from molecular dynamics simulations. *Biochimica et Biophysica Acta (BBA) - Biomembranes* **1858,** 2390–2400 (2016).
5. Humphrey, W., Dalke, A. & Schulten, K. VMD: Visual molecular dynamics. *Journal of Molecular Graphics* **14,** 33–38 (1996).
6. Michaud-Agrawal, N., Denning, E. J., Woolf, T. B. & Beckstein, O. MDAnalysis: a toolkit for the analysis of molecular dynamics simulations. *J. Comput. Chem.* **32,** 2319–2327 (2011).
7. Gowers, R. J. *et al.* MDAnalysis: A Python package for the rapid analysis of molecular dynamics simulations. in (eds. Benthall, S. & Rostrup, S.) 102–109 (Wiley Subscription Services, Inc., A Wiley Company, 2016). doi:10.1002/jcc.21787
8. Schrödinger, LLC. The PyMOL Molecular Graphics System, Version 1.7.0.0. (2010).
